# Psychotic symptoms and suicidality: combined findings from a high-risk and a clinical cohort of adolescents

**DOI:** 10.1007/s00787-026-02990-z

**Published:** 2026-03-25

**Authors:** Diandra C. Bouter, Arianna Vecchio, Marika Orlandi, Nita G.M. de Neve-Enthoven, Witte J.G. Hoogendijk, Renato Borgatti, Nina H. Grootendorst-van Mil, Martina M. Mensi

**Affiliations:** 1 Department of Psychiatry, Erasmus MC, University Medical Center Rotterdam, Rotterdam, the Netherlands; 2 Child Neurology and Psychiatry Unit, IRCCS Mondino Foundation, Pavia, Italy; 3 Department of Brain and Behavioral Sciences, University of Pavia, Pavia, Italy; 4 Epidemiological and Social Psychiatric Research Institute (ESPRi), Department of Psychiatry, Erasmus MC, University Medical Center Rotterdam, Rotterdam, the Netherlands

**Keywords:** Epidemiology, Psychiatry, Suicidality, Psychotic symptoms, Adolescents

## Abstract

Suicidality is common across the psychosis spectrum. To better understand this relationship, studies across different settings, varying levels of severity, and in-depth examination of psychotic symptoms, are needed. Here, we examined the association between psychotic symptoms and suicidality in adolescents who are at high-risk for psychopathology or already engaged in mental health care. Data were combined from a high-risk cohort from the general population (*n* = 156, 49.4% female, mean age 18.7 years) and a clinical sample receiving mental health care (*n* = 95, 86.3% females, mean age 15.2 years). Adolescents were interviewed on unusual thought content, non-bizarre delusional ideas, or hallucinations and assessed on suicidal ideations (SI) and suicide attempts (SA). Co-occurring psychopathology was measured with a semi-structured DSM-5 interview. Among the 251 adolescents, 50 (19.9%) reported SI and 59 (23.5%) also reported SA. When examined separately, all types of psychotic symptoms were associated with SI, SA, and suicidality severity. Multinomial regression analysis showed that unusual thought content was associated with SI (age and sex adjusted OR = 8.05 [2.79;23.19]). Both non-bizarre delusional ideas and unusual thought content were associated with a higher likelihood of SA (OR = 3.89 [1.37;11.04] and OR 6.45 [2.30;18.08], respectively). Notably, hallucinations were not associated with suicidality when delusional ideas were simultaneously considered. Results were independent of co-occurring psychopathology and multimorbidity (i.e. the presence of multiple disorders). Psychotic symptoms, especially delusional ideas, were associated with suicidality in adolescents. Psychotic symptoms may serve as important markers for suicidality, underscoring the need for closer monitoring in at-risk youth.

**Supplementary Information:**

The online version contains supplementary material available at 10.1007/s00787-026-02990-z.

## Introduction

Suicidality is prevalent in adolescents, 10–20% of adolescents have considered suicide and 7–10% have attempted suicide [1–3]. An alarming number of adolescents die by suicide, making it one of the leading causes of death in youth [4]. Suicidality includes both suicidal ideation (SI), i.e. thinking about or planning suicide, as well as suicide attempt (SA) [5]. In a meta-analysis of 13 large register studies, psychotic disorders showed the highest risk for suicide compared to other psychiatric diagnoses [6]. All disorders within the psychosis spectrum have been linked to SA, multiple attempts, and suicide death [7–10].

To better understand the association between psychotic disorders and suicidality, previous studies have examined the precursors of psychotic disorders by studying psychotic experiences (PE). In childhood, delusional experiences, dissociative experiences, and auditory hallucinations were associated with SI and SA [11, 12]. Another study found that specifically childhood hallucinatory experiences were associated with suicidality in adolescence [12, 13]. In adolescents, auditory hallucinatory experiences but not delusional experiences were associated with SA over time [14]. Auditory hallucinations were also linked to the transition from ideation to attempt [15]. In help-seeking adolescents, PE were associated with suicide plans and attempts [16]. A meta-analysis on over 84,000 participants showed that PE were associated with future SI and SA in the general population, even after adjustment for co-occurring psychopathology [17]. These studies focused on PE, a potential precursor of psychotic disorders, but PE are often transient and not progressive [18–20].

Although studies have consistently demonstrated a strong association between the psychosis spectrum and suicidality, current research remains inconclusive as most studies focused on the extremes of the psychosis spectrum by examining either psychotic experiences or psychotic disorders. This is further complicated by the varying terminology used for different characteristics of the psychosis spectrum. It is important to acknowledge that different terms can reflect different populations. Psychotic experiences are often studied in the general population, attenuated psychotic symptoms in at-risk or prodromal participants, and psychotic symptoms in all types of populations including patients with a psychosis spectrum disorder, each populations with their own symptom profile and risk trajectory [21]. Recent (umbrella) meta-analyses have not identified psychotic symptoms as an established risk factor for suicidality in adolescents [22, 23]. However, findings from a two-cohort study in adolescents showed that psychotic symptoms were associated with a clear increased risk of suicidal behavior [24]. In adolescents and young adults at clinical high-risk of psychosis (CHR) it was shown that especially positive symptoms were linked to suicidality [25]. Both highlighting the need for further investigation. As recently written in a call to action, more studies are necessary with varying levels within the spectrum of psychosis and suicidality, studied in participants from different settings [26]. Here, we present combined findings from a cohort that include adolescents from the general population who were at high-risk for psychopathology and a cohort of adolescents receiving mental health care for psychopathology. We specifically examined clinician-rated psychotic symptoms, measuring both hallucinations and delusions in these adolescents. The combination of the two cohorts enabled us to study a broad range of psychotic symptoms and varying degrees of suicidality. The objective was to study the association between psychotic symptoms and suicidality in adolescents.

## Methods

### Participants

To study the spectrum of both psychotic symptoms and suicidality, we combined data from two cohorts of adolescents who underwent detailed assessment for psychotic symptoms and suicidality. One cohort included adolescents at high-risk of psychopathology and the other cohort included those who were already receiving treatment for psychiatric problems.

The high-risk adolescents were selected from a population-based high-risk cohort from the Netherlands. These adolescents were considered at high risk of psychopathology as they were screened at secondary schools at age 13 for emotional and behavioral problems with the Strength and Difficulties Questionnaire – Youth) and showed elevated levels of difficulties at age 15 [27]. High-risk adolescents were selected based on scoring in the highest 15% on the questionnaire, low-risk adolescents were randomly selected from the lowest 85% on the questionnaire. During follow-up at age 18 (79% response rate), it was demonstrated that selecting high-risk adolescents based on this early screening was associated with significantly higher levels of psychopathology, with an up to a sevenfold increased risk of developing a psychiatric disorder compared to low-risk adolescents [28]. In this study, “risk” was defined in a broader, transdiagnostic sense, reflecting vulnerability to various forms of psychopathology rather than being specific to psychosis. The study was approved by the Medical Research Ethics Committee of the Erasmus Medical Center Rotterdam and participants were included after providing written informed consent. Approval from their legal guardians was obtained for adolescents under the age of 16, in line with local laws and regulations. Data were collected between March 2019 and June 2022. Additional information on the high-risk cohort can be found in the study design article [27].

The clinical cohort comprised adolescents who were receiving treatment at the Child Neurology and Psychiatry Unit of the IRCCS Mondino Foundation in Pavia, Italy. All adolescents were aged between 12 and 18 years and referred to this tertiary healthcare center by either secondary mental healthcare services or local primary care. Adolescents were admitted to the Child Neuropsychiatry unit as either outpatients or inpatients for various psychiatric problems. Patients were included into the presented study if they completed assessments on both psychotic symptoms and suicidality. The study was approved by the Medical Research Ethics Committee of the Policlinico San Matteo in Pavia. Written informed consent was given by all adolescents and their legal guardians. Data were collected between March 2019 and January 2023.

### Measurements

#### Psychotic symptoms

To combine data from the cohorts, we integrated data from two separate measurements to assess psychotic symptoms. In the high-risk cohort, psychotic symptoms were assessed with the semi-structed Mini Neuropsychiatric Interview for Children and Adolescents (MINI KID [29, 30]), . A trained clinician rated whether the adolescent had experienced delusions or hallucinations. Delusions included persecutory ideas, mind reading, idea of being controlled, receiving special messages, and other delusional ideas. Hallucinations included both auditory and visual perceptual abnormalities.

In the clinical cohort, subscales from the Comprehensive Assessment of At Risk Mental States (CAARMS [31]), were used. Trained psychologists assessed adolescents on positive symptoms in three subscales: unusual thought content, non-bizarre delusional ideas, and perceptual abnormalities. Based on the cut-offs scores of the CAARMS, a severity rating of three or higher was used to select adolescents with psychotic symptoms [31]. This cut-off score identifies adolescents who experience psychotic symptoms, but these symptoms do not need to meet the severity or frequency threshold for the psychotic episode cut-off. In the clinical cohort, 72.5% of the adolescents reported one or more psychotic symptoms (CAARMS score of ≥ 3), and 7.2% of these adolescents met the severity rating of severe psychotic symptoms (CAARMS score of 6).

The items of the MINI KID were divided across the three dichotomized CAARMS scales, where unusual thought content included ideas of feeling controlled, reading of thoughts, and ideas of reference. Non-bizarre delusional ideas included suspicious ideas, persecutory thoughts and ideas such as grandiosity, severe guilt or jealousy.

Combination of the two measurements was based on discussions between clinicians and researchers from both sites who collected the data. A detailed description of the items used and how they were combined is available in the supplementary materials.

#### Suicidality

The Columbia-Suicide Severity Rating Scale (C-SSRS [32]), was used to measure suicidality in both cohorts. Adolescents were interviewed to assess SI and SA. SI included five items, with increasing severity, to assess the wish to be dead, active suicidal thoughts, specificity of suicide plans, and the intent to act. SA were assessed in three items to measure aborted, interrupted, or actual suicide attempts. Severity of the suicidal behavior was assessed by the type of SI and the number of SA.

#### Psychopathology and other covariates

Psychopathology was assessed with semi-structured interviews for DSM-5 psychiatric disorders diagnoses, either the MINI KID (population-based high risk cohort) [30] or the Kiddie Schedule for Affective Disorders and Schizophrenia (clinical setting) (KSADS [33]), . A total of 18 diagnoses were assessed in the cohorts: depressive disorder, bipolar disorder, panic disorder, agoraphobia, separation anxiety, social phobia, specific phobia, general anxiety, obsessive-compulsive disorder (OCD), post-traumatic stress disorder (PTSD), anorexia nervosa, bulimia nervosa, binge eating disorder, attention deficit hyperactivity disorder (ADHD), oppositional defiant disorder (ODD), conduct disorder (CD), alcohol use, and substance use disorders.

The Children’s Global Assessment Scale (CGAS, clinical cohort) and Global Assessment of Functioning (GAF, high-risk cohort) were used to measure the global functioning of the adolescents [34, 35]. In the population-based high-risk cohort an estimation of IQ was obtained with the shortened SON-R non-verbal IQ test (SON-R [36]). In the clinical cohort, adolescents under 16 years were assessed with the Wechsler Intelligence Scale for Children (WISC-IV [37]) and those 16 years or older completed the Wechsler Adult Intelligence Scale (WAIS-IV [38]).

### Statistical analyses

First, descriptive statistics were calculated. Pearson correlations between psychotic symptoms were obtained and interpreted according to Cohen’s guidelines. Differences in suicidality across various psychotic symptoms were compared using Chi-square tests. Next, we examined whether severity of suicidality ( ideation severity, number of suicide attempts) was associated with psychotic symptoms using Chi-square tests. Multinomial regression analyses were used to assess whether psychotic symptoms were associated with SI an SA. The analysis was adjusted for age (rounded) and sex, with the absence of suicidality serving as the reference category. Next, we examined whether the association between psychotic symptoms and suicidality was independent of co-occurring psychopathology. In separate analyses we additionally adjusted for internalizing disorders (mood, anxiety and PTSD, OCD, and eating disorders), externalizing disorders (ADHD, disruptive disorders, and substance use disorders) or for multimorbidity (number of diagnoses). Finally, to test the robustness of our results we added interaction terms to the models to examine potential sex-interaction effects as well as interaction effects between delusions and hallucinations. Adolescents were included based on the availability of data on both psychotic symptoms and suicidality. There were no missing values for the covariates with the exception of co-occurring psychopathology (*n* = 3, 1.2%), these three adolescents were excluded from those analyses. All analyses were performed using IBM SPSS Statistics 29.0 [39].

#### Sensitivity analyses

To ensure results were comparable and not driven by the characteristics of a specific cohort, differences in suicidality across various psychotic symptoms were examined with Fisher’s exact test and the multinomial regression analysis were examined separately in each cohort.

## Results

A total of 251 adolescents were included based on the availability of suicidality and psychotic symptoms measures. Of these adolescents, 156 (62.2%) were from the Dutch high-risk cohort and 95 (37.8%) were from the Italian clinical cohort. The demographic and clinical characteristics of the total cohort are described in Table 1. Adolescents had a mean age of 17.6 years, and 63.3% adolescents were girls. Suicidality was reported by 109 adolescents (43.4%), 50 adolescents reported SI without SA(19.9%), and 59 adolescents reported both SI and SA (23.5%). The characteristics of the high-risk and clinical adolescents separately are described in Supplementary Table S1. The high-risk cohort consisted of 77 females (49.4%) with a mean age of 18.7 years (SD = 0.68) and the clinical cohort consisted of 82 females (86.3%) with a mean age of 15.2 years (SD = 1.56). As expected, the two cohorts differed in characteristics given the different sampling strategy used to obtain the cohorts. The clinical cohort had lower global functioning scores (Mdn = 50, IQR 40–60) compared to the high risk cohort (Mdn = 65, IQR 55–75).

**Table 1:** Characteristics, psychotic symptoms, and psychopathology of the included adolescents

	Adolescents	
	*n* = 251	
	*n*	%*
Sex, *female*	159	63.3
Age *(M*,* SD)*,* years*	17.59	1.35
Estimated IQ *(M*,* SD)*	100.77	14.30
Global assessment of functioning *(Median*,* IQR)*	60	50–71
Psychotic symptoms		
Delusions, non-bizarre ideas	45	17.9
Delusions, unusual thoughts	62	24.7
Hallucinations, perceptual abnormalities	64	25.5
Psychopathology		
Mood disorders	107	43.1
Anxiety disorders	76	30.6
Obsessive-compulsive disorders	13	5.2
Post-traumatic stress disorder	9	3.7
Substance use disorders	78	31.5
ADHD	37	14.9
Disruptive behavior disorders	26	10.5
Eating disorders	29	11.7

Note. Diagnoses were based on a semi-structed DSM-5 interview which was missing for 3 adolescents, IQ scores were missing for 21 adolescents.

*Numbers represent % unless otherwise indicated

A total of 94 adolescents (37.5%) reported psychotic symptoms. The two delusional ideas scales were moderately correlated (*r*=.50, *p*<.001), hallucinations were only weakly correlated with non-bizarre delusional ideas (*r*=.39, *p*<.001) and moderately correlated with the unusual thought scale (*r*=.51, *p*<.001). Most adolescents who reported hallucinations also reported any type of delusional symptom (70.3%).

Psychopathology was prevalent in most adolescents, only 22.2% of the adolescents did not meet the criteria for any diagnosis. About 29.8% adolescents met the criteria for one diagnosis, with the remaining adolescents had multimorbidity, meeting the criteria for two, three, or ≥four diagnoses (20.2%, 11.3%, and 16.5%, respectively).

### Psychotic symptoms and suicidality

First, chi-square tests were used to examine the association between the presence of psychotic symptoms and suicidality (Table 2). Non-bizarre delusional ideas, unusual thought content, and perceptual abnormalities were all associated with SI and SA.

**Table 2:** Differences in psychotic symptoms and reported suicidality in 251 adolescents

	Suicidality						
	*None* *n* = 142		*Ideationsn* = 50		*Attemptsn* = 59		
	*n*	%	*n*	%	*n*	%	
Delusions, non-bizarre ideas							***Χ***^**2**^ **= 39.80**, ***p*<.001**
absent	134	65.0	38	18.4	34	16.5*	
present	8	17.8**	12	26.1	25	55.6**	
Delusions, unusual thought content							***Χ***^**2**^ **= 65.44**, ***p*<.001**
absent	134	70.9**	28	14.8	27	14.3**	
present	8	12.9***	22	35.5	32	51.6***	
Hallucinations, perceptual abnormalities							***Χ***^**2**^ **= 33.36**, ***p*<.001**
absent	124	66.3	34	18.2	29	15.5*	
present	18	28.1**	16	25.0	30	46.9***	

Note. Based on the standardized residual (z-score) significant at * *p*<.05, ** *p*<.01, or *** *p*<.001

Next, we examined whether the adolescents who reported psychotic symptoms more often reported severe suicidality (supplementary table S2). Adolescents with non-bizarre delusional ideas, unusual thoughts, and perceptual abnormalities were more likely to report active SI with a plan and intent to act as well as to have conducted one or multiple SA.

Third, we examined the association between psychotic symptoms and SI and SA in multinomial regression analyses (Table 3). The absence of suicidality was used as the reference category. An association was found between unusual thought content and both SI and SA. Non-bizarre delusional ideas were associated with SA only, whereas perceptual abnormalities were not associated with either SI or SA.

**Table 3:** Associations between psychotic symptoms and suicidality (*n* = 251)

		OR	95% CI		*p*-value
Suicidal ideation vs. no suicidality					
Intercept					0.60
Sex (female)		1.21	0.57	2.59	0.62
Age		0.86	0.70	1.05	0.14
Delusions, non-bizarre ideas		1.85	0.60	5.70	0.29
Delusions, unusual thoughts		**8.05**	**2.79**	**23.24**	**< 0.001**
Hallucinations, perceptual abnormalities		0.92	0.34	2.51	0.88
Suicide attempt vs. no suicidality					
Intercept					0.49
Sex (female)		**3.90**	**1.54**	**9.93**	**0.00**
Age		0.93	0.75	1.14	0.48
Delusions, non-bizarre ideas		**3.86**	**1.36**	**10.92**	**0.01**
Delusions, unusual thoughts		**6.47**	**2.30**	**18.17**	**< 0.001**
Hallucinations, perceptual abnormalities		1.66	0.63	4.38	0.30

Note. Absence of suicidality was the reference category

When the psychotic symptoms were examined in individual multinomial regression models, only the delusional scales were associated with SI (OR_non−bizarre_ = 3.85 [1.42;10.46], and OR_unusual_ = 9.49 [3.59;25.05]). All type of psychotic symptoms were individually associated with SA (OR_non−bizarre_ = 8.35 [3.27;21.35]; OR_unusual_ = 11.85 [4.59;30.61], and OR_hallucinations_=3.98 [1.76;9.00]).

### Co-occurring psychopathology

We examined whether the associations were independent of comorbid psychopathology. In three analyses we adjusted for internalizing disorders (mood, anxiety and PTSD, OCD, and eating disorders), externalizing disorders (ADHD, ODD, CD and substance use disorders), or multimorbidity (number of diagnoses). The results are presented in Table 4.

**Table 4:** Coefficients from multinomial regression analyses for the associations between psychotic symptoms and suicidality, after additional adjustment for co-occurring psychopathology (*n* = 248)

	Additionally adjusted for internalizing disorders				Additionally adjusted for externalizing disorders						Additionally adjusted for multimorbidity			
	OR	95% CI		*p*		OR	95% CI		*p*		OR	95% CI		*p*
Suicidal ideation vs. no suicidality														
Intercept				0.35					0.29					0.46
Sex (female)	0.95	0.41	2.21	0.90		1.22	0.55	2.70	0.63		1.18	0.54	2.59	0.68
Age	0.80	0.64	1.00	0.06		0.80	0.64	1.01	0.06		0.81	0.65	1.01	0.06
Delusions, non-bizarre ideas	1.72	0.49	5.99	0.40		1.92	0.58	6.36	0.29		1.83	0.55	6.02	0.32
Delusions, unusual thoughts	**6.84**	**2.14**	**21.89**	**< 0.001**		**7.91**	**2.64**	**23.65**	**< 0.001**		**8.07**	**2.67**	**24.44**	**< 0.001**
Hallucinations, perceptual abnormalities	0.77	0.26	2.30	0.65		0.93	0.33	2.59	0.88		0.91	0.32	2.54	0.85
Mood disorders	**4.38**	**1.96**	**9.77**	**< 0.001**	ADHD	0.88	0.36	2.14	0.78	1 diagnosis	2.00	0.70	5.70	0.19
Anxiety and PTSS	0.79	0.33	1.92	0.60	Disruptive disorders	1.25	0.41	3.80	0.70	2 diagnoses	1.61	0.49	5.34	0.43
OCD	1.36	0.22	8.34	0.74	Substance use disorders	1.76	0.41	7.54	0.44	3 diagnoses	2.67	0.73	9.82	0.14
Eating disorders	0.82	0.22	2.99	0.76						≥ 4 diagnoses	2.19	0.62	7.73	0.22
Suicide attempt vs. no suicidality														
Intercept				0.73					0.66					0.72
Sex (female)	2.76	0.98	7.71	0.05		**3.28**	**1.26**	**8.57**	**0.02**		**3.76**	**1.44**	**9.82**	**0.01**
Age	0.86	0.68	1.09	0.22		0.92	0.72	1.16	0.47		0.85	0.68	1.07	0.16
Delusions, non-bizarre ideas	2.95	0.91	9.56	0.07		**4.69**	**1.52**	**14.50**	**0.01**		**3.49**	**1.16**	**10.56**	**0.03**
Delusions, unusual thoughts	**5.49**	**1.71**	**17.70**	**< 0.001**		**7.10**	**2.41**	**20.90**	**< 0.001**		**5.74**	**1.92**	**17.17**	**< 0.001**
Hallucinations, perceptual abnormalities	1.57	0.53	4.65	0.42		1.69	0.62	4.62	0.31		1.68	0.62	4.56	0.31
Mood disorders	**6.29**	**2.64**	**14.98**	**< 0.001**	ADHD	0.48	0.17	1.33	0.16	1 diagnosis	3.04	0.86	10.75	0.08
Anxiety and PTSS	1.99	0.84	4.70	0.12	Disruptive disorders	2.11	0.68	6.55	0.20	2 diagnoses	2.91	0.76	11.06	0.12
OCD	0.89	0.14	5.70	0.90	Substance use disorders	0.65	0.11	4.05	0.65	3 diagnoses	3.63	0.82	16.15	0.09
Eating disorders	0.62	0.17	2.28	0.48						≥ 4diagnoses	**5.99**	**1.54**	**23.22**	**0.01**

Note. PTSS Posttraumatic Stress Disorder, OCD Obsessive-Compulsive Disorder, ADHD Attention-Deficit Hyperactivity Disorder

Adding mood disorders to the model attenuated the association between unusual thoughts and SI. After additional adjustment for mood disorders the association between non-bizarre delusional ideas and SA was no longer significant, the association with unusual thoughts was slightly attenuated. The association between psychotic symptoms and SI or SA was independent of all other types of psychopathology. Multimorbidity was associated with SA (meeting the criteria for ≥ 4 diagnoses, similar OR’s were found after additional adjustment for multimorbidity.

### Interaction effects

To test the robustness of our results we added two interaction terms to the model. First, we examined the interaction between sex and psychotic symptoms. Next, we examined the interaction between delusions and hallucinations. As shown in supplementary table S3 no significant interactions were found for sex or between the type of psychotic symptoms.

### Sensitivity analyses

The present study combined a high-risk cohort and a clinical cohort of adolescents, each with distinctive characteristics. Stratified by cohort analyses were performed to examine whether the associations were consistent across the two cohorts rather than specific to one of the two cohorts. Results of the stratified analyses are presented in Supplementary Table S4 and S5 and showed similar associations in the two cohorts. The only discrepancy was that we did not find an association between non-bizarre delusional ideas and SA in the clinical cohort.

## Discussion

In our combined two-site cohort of adolescents, we studied the associations between psychotic symptoms and suicidality, specifically SI and SA. These adolescents showed high levels of psychopathology, with 78% of the adolescents meeting the criteria for one or multiple DSM-diagnoses, and around 25% of the adolescents reporting delusions or hallucinations. When investigated separately, we found that all types of psychotic symptoms were associated with SI, SA, as well as suicidality severity. However, when considering the type of symptoms simultaneously, we found that unusual thought content was associated with both SI and SA and non-bizarre delusional ideas were only associated with SA. Notably, we did not find an association between perceptual abnormalities and suicidality when taking delusional ideas into account. For unusual thought content these associations were independent of co-occurring psychopathology or multimorbidity. The association between non-bizarre delusional ideas and SA was independent of multimorbidity but dependent on mood disorders.

In line with previous studies we showed that both delusions and hallucinations were individually associated with suicidality and more severe suicidal behavior [11–14, 16, 17, 24]. In contrast to previous studies, we did not find a robust association with perceptual abnormalities and suicidality. Previously auditory and visual hallucinations were shown to be associated with suicidality [13, 14]. In schizophrenia patients, meta-analyses showed contrasting results with regard to hallucinations, hallucinations showed both a reduced and a small increased risk of suicide, as a result of heterogeneity of the data [40, 41]. A potential explanation could be that previous studies did not simultaneously take delusional experiences into account, although psychotic symptoms often consist of distinct dimensions which are only moderately correlated [42]. Some studies only examined hallucinations and not delusions, thereby focusing on a specific symptom subtype [43]. To understand the etiology of delusions, a comprehensive theoretical model describes that anomalous experiences, such as hallucinations, precede delusional beliefs [44]. In our sample we had similar prevalences of hallucinations and delusions, the majority of the participating adolescents with hallucinations also experienced delusions, thus masking the slightly smaller effect of hallucinations found. On the other hand, it could also be that especially the adolescents who experienced both hallucinations and delusions were more likely to report suicidality, as the co-occurrence of these symptoms is associated with high clinical severity [45], which could not be distinguished in our multinomial regression model.

We specifically found that delusional symptoms, and not hallucinations, were associated with increased suicidality. This association was also found for delusional experiences in childhood [11, 12] whereas in adolescents contrasting results were found [13, 14]. In schizophrenia patients, delusions have been associated with an elevated risk of suicide [41]. As fewer studies specifically focused on delusional ideas, it is interesting to consider potential mechanisms underlying this association. Although we could not examine this in our combined cohort, it is possible that distress associated with the delusional symptoms could be an underlying mechanism resulting in suicidality [11, 42, 46]. In adolescents and young adults from the general population delusions were strongly linked to distress, depression, and poor functioning [47]. In psychosis patients, distress caused by delusional symptoms was linked to SI [48, 49]. It could be that the distress associated with the delusions resulted in suicidality in these adolescents [47–50], which could align with a recent review that implied distress as a potential treatment target [51]. We showed that these associations were independent of multimorbidity, but as expected not all associations were independent of co-occurring mood disorders. These mood symptoms could be an indication of co-occurring negative symptoms as there is conceptual overlap between negative symptoms and depressive symptoms [52–54]. More research is necessary to determine if depressive symptoms are a confounder or a mediator in the association between psychotic symptoms and suicidality [55, 56].

It is important to underline that the discussed studies on psychosis and suicidality all examined various variations within the psychosis spectrum. Specifically for the current study we defined psychotic symptoms as clinician-rated hallucinations and delusions, but did not take into account the frequency or distress associated with these symptoms. There are distinct differences between full-threshold psychotic symptoms, attenuated psychotic symptoms, or psychotic experiences, as well as between individuals who are administered a simple psychosis-risk screening tool compared to those identified as CHR. These constructs differ both in terms of symptom severity as well as in distress or help-seeking behavior [21]. Each construct has its own symptom profile and risk trajectory which should be considered when discussing the associations between specific levels of psychotic symptoms and suicidality. Moreover, not only the type of psychotic construct, also the type of measurement should be considered. For instance, self-reported measurements tend to overestimate the prevalence compared to clinician-rated symptoms, even though both have been associated with later risk for psychiatric disorders [57–59].

The study had some limitations that should be addressed. First, data on psychotic symptoms and suicidality were collected at a single time point, making it not possible to examine the direction of the association and bidirectional effects that have been found in adolescents [60]. However, most previous studies have found that psychotic experiences precede suicidality, which underlines the suicidal drive hypothesis, i.e. the externalization of psychotic symptoms into suicidal behavior [17, 61–63]. Second, because of relatively small numbers of SI and SA, as well as challenges in harmonizing additional data between the cohorts, our analyses were not adjusted for other potentially relevant factors besides age, sex and psychopathology. Nonetheless, a systematic review and several recent studies showed that in most studies the associations remained after adjustment for other variables, indicating an independent association between psychotic symptoms and suicidality [56, 64, 65]. Third, there was no data available on the specific content of the delusions. Therefore we could not conclude whether the content of the psychotic symptoms was linked to suicidality or that these delusions might be an indication of severe psychopathology associated with suicidality. Given that the association remained after adjustment for co-occurring psychopathology it seems that the specific delusional thought content is associated with suicidality in adolescents. Finally, our study combined two cohort with different characteristics. Although we adjusted our analyses for differences between the two samples (e.g. sex, age, psychopathology) and found similar associations in the stratified analyses, it is necessary to consider this in terms of generalizability and comparability of the findings. Future research should further explore the potential influence of key characteristics of the adolescents by studying diverse samples and settings to further validate and extend the current findings. Of particular interest are characteristics such as age, sex, substance use and co-occurring psychopathology, as previous studies did find these characteristics to be associated [66–72]. In particular, more research is necessary to examine the severity of the psychotic symptoms as we could only study the presence or absence of specific psychotic symptoms without examining the severity in terms of frequency, distress or help-seeking behavior.

In turn, there are also strengths that should be considered. We combined two rich datasets with data from a population-based high-risk cohort and help-seeking adolescents. This made it possible to study the full spectrum of psychotic symptoms and suicidality. Furthermore, all data was collected by trained clinicians and researchers. By using interview-based data on both psychotic symptoms and suicidality we were able to study these associations in detail. Especially the C-SSRS is a very detailed interview to assess suicidality, to capture both suicidal ideation and suicide attempt, while also allowing for the measurement of their severity. Finally, the detailed data made it possible to see if the associations were independent of co-occurring psychopathology by taking into account the presence of internalizing disorders, externalizing disorders and multimorbidity in these adolescents.

This study showed that psychotic symptoms, especially delusional ideas, were associated with suicidality in adolescents. Our findings underline the importance of considering the different dimensions of psychotic symptoms by separating delusions and hallucinations. Most previous research has focused on the extremes of the psychosis continuum, either examining psychotic experiences or full-blown psychosis. Here, we added that psychotic symptoms were associated with SI and SA in adolescence, independent of their help-seeking behavior. Importantly, our findings emphasize the need for early detection and intervention, as psychotic symptoms may serve as an important indicator of suicidality risk. This provides possibilities for screening and targeted prevention by assessing psychotic symptoms and the associated suicidality risk in adolescence.

## Supplementary Information

Below is the link to the electronic supplementary material.

Supplementary Material 1

## Data Availability

Researchers are welcome to collaborate and request access to the data. Proposals will be discussed with the project team to assess quality, feasibility, and potential overlap with planned or published publications.
