# Supplementary material for: Psychotic symptoms and suicidality: combined findings from a high-risk and a clinical cohort of adolescents
Source: Eur Child Adolesc Psychiatry. 2026 Mar 25;35(6):2031–40. doi: 10.1007/s00787-026-02990-z (PMC13337856; doi:10.1007/s00787-026-02990-z)
Supplement: Supplementary file 1 — Supplementary Material 1 [file 787_2026_2990_MOESM1_ESM.pdf]

Supplementary material

Psychotic symptoms and suicidality: combined findings from a high-risk and a clinical cohort of adolescents

Diandra C. Bouter, Arianna Vecchio, Marika Orlandi, Nita G.M. de Neve-Enthoven, Witte J.G. Hoogendijk, Renato Borgatti, Nina H. Grootendorst-van Mil, Martina M. Mensi

Measurement of psychotic symptoms

Clinical cohort - Comprehensive Assessment of At-Risk Mental States (CAARMS) as designed by
Yung, A.R., et al., *Mapping the onset of psychosis: the Comprehensive Assessment of At-Risk Mental States.* Aust N Z J Psychiatry, 2005. **39**(11-12): p. 964-71.

	**0**<br>**Never, absent**	**1**<br>**Questionable**	**2**<br>**Mild**	**3**<br>**Moderate**	**4**<br>**Moderately severe**	**5**<br>**Severe**	**6**<br>**Psychotic and Severe**
Unusual Thought Content – Global Rating Scale	No Unusual Thought Content.	Mild elaboration of Conventional beliefs as held by a proportion of the population	Vague sense that something is different, or not quite right with the world, a sense that things have changed but not able to be clearly articulated.<br><br>Subject not concerned/ worried about this experience	A feeling of perplexity.<br>A stronger sense of uncertainty regarding thoughts than 2.	Referential ideas that certain events, objects or people have a particular and unusual significance.<br><br>Feeling that experience may be coming from outside the self. Belief not held with conviction, subject able to question. Does not result in change in behaviour.	Unusual thoughts that contain completely original and highly improbable material.<br><br>Subject can doubt (not held with delusional conviction), or which the subject does not believe all the time.<br><br>May result in some change in behaviour, but minor.	Unusual thoughts Containing original and highly improbable material held with delusional conviction (no doubt).<br><br>May have marked impact on behaviour.
Non-bizarre Ideas – Global Rating Scale	No Non-bizarre Ideas.	Subtle changes that could be reality	Increased self-conscious-ness e.g.	Odd or unusual thoughts but whose content	Clearly idiosyncratic beliefs, which	Unusual thoughts about which there is	Unusual thoughts Containing original

		based e.g. very self-conscious.	feeling that others look at the subject, or talk about the subject.<br><br>Or feeling of increased self-importance. Subject able to question.	is not entirely implausible- may be some logical evidence. More evidence than rating of 4.<br><br>Content of thoughts not original i.e. jealousy, mild paranoia.	although 'possible' have arisen without logical evidence.<br><br>Less evidence than rating of 3 e.g. thoughts that others wish the subject harm, which can be easily dismissed.<br><br>Thoughts of having special powers, which can be easily dismissed.	some doubt (not held with delusional conviction), or which the subject does not believe all the time.<br><br>May result in some change in behaviour, but minor.	and highly improbable material held with delusional conviction (no doubt).<br><br>May have marked impact on behaviour.
Perceptual Abnormalities – Global Rating Scale	No abnormal perceptual experience.		Heightened, or dulled perceptions, distortions, illusions (e.g. lights/ shadows). Not particularly distressing. Hypnogogic/ hypnopompic experiences	More puzzling experiences: more intense/vivid distortions/illusions, indistinct murmuring, etc.<br><br>Subject unsure of nature of experiences.<br><br>Able to dismiss.<br><br>Not distressing.<br><br>Derealisation/ depersonalisation	Much clearer experiences than 3 such as name being called, hearing phone ringing etc. but may be fleeting/ transient.<br><br>Able to give plausible explanation for experience. May be associated with mild distress.	True hallucinations i.e. hearing voices or conversation, feeling something touching body.<br><br>Subject able to question experience with effort.<br><br>May be frightening or associated with some distress.	True hallucinations which the subject believes are true at the time of, and after,experiencing them.<br><br>May be very distressing

High-risk cohort – MINI KID as designed by
Sheehan, D.V., et al., *Reliability and validity of the Mini International Neuropsychiatric Interview for Children and Adolescents (MINI-KID)*. Journal of Clinical Psychiatry, 2010. **71**(3): p. 313-26.

7 items scored as either yes or no were divided over the following three CAARMS scales:

<u>Unusual thought content</u>
Have you ever believed that someone was reading your mind or could hear your thoughts, or that you could actually read someone's mind or hear what another person was thinking?

Have you ever believed that someone or some force outside of yourself put thoughts in your mind that were not your own, or made you act in a way that was not your usual self? Have you ever felt that you were possessed?

Have you ever believed that you were being sent special messages through the TV, radio, internet, newspapers, books, or magazines or that a person you did not personally know was particularly interested in you?

<u>Non-bizarre ideas</u>
Have you believed that people were spying on you, or that someone was plotting against you, or trying to hurt you?

Have your relatives or friends ever considered any of your beliefs odd or unusual?

<u>Perceptual abnormalities</u>
Have you ever heard things other people couldn't hear, such as voices?

Have you ever had visions when you were awake or have you ever seen things other people couldn't see?

Supplementary table S1. Characteristics of the included adolescents, stratified by cohort

	**High-risk cohort (*n*=156)**		**Clinical cohort (*n*=95)**		
	n	%*	*n*	%*	
Sex, *female*	77	49.4	82	86.3	**$\chi2$ (1) = 34.73, *p* <.001**
Age *(M, SD)*	18.7	0.68	15.2	1.56	***t* (116) = 20.76, *p* <.001**
Estimated IQ *(M, SD)*	98.04	12.79	105.08	15.53	***t* (161) =-3.74, *p* <.001**
Global Assessment of Functioning *(Median, IQR)*	65	55-75	50	40-60	***U* =2456.5, *p* <.001**
Psychotic symptoms					
Delusions, non-bizarre ideas	12	7.7	33	34.7	**$\chi2$ (1) =29.35, *p* <.001**
Delusions, unusual thought content	10	6.4	52	54.7	**$\chi2$ (1) = 74.14, *p* <.001**
Hallucinations, perceptual abnormalities	11	6.4	53	55.8	**$\chi2$ (1) = 73.83, *p* <.001**
Psychopathology					
Mood disorders	50	32.1	57	62	**$\chi2$ (1) = 21.10, *p p* <.001**
Anxiety disorders	39	25.0	37	40.2	**$\chi2$ (1) = 6.31, *p* = .012**
Obsessive-compulsive disorders	6	3.8	7	7.6	$\chi2$ (1) = 1.65, *p* = .199
Post-traumatic stress disorder	7	4.5	2	2.2	$\chi2$ (1) = .83, *p* = .362
Substance use disorders	73	46.8	5	5.4	**$\chi2$ (1) = 45.92, *p* <.001**
ADHD	16	10.3	3	3.3	**$\chi2$ (1) = 15.66, *p* <.001**
Disruptive behavior disorders	16	10.3	10	10.9	**$\chi2$ (1) = 4.00, *p* = .045**
Eating disorders	4	2.6	25	27.2	**$\chi2$ (1) = 33.94, *p* <.001**
Multimorbidity					$\chi2$ (4) = 6.75, *p* =.150
No diagnosis	38	24.4	17	18.5	
1 diagnosis	51	32.7	23	25.0	
2 diagnoses	26	16.7	24	26.1	
3 diagnoses	14	9.0	14	15.2	
4 or more diagnoses	27	17.3	14	15.2	

Note. Diagnoses are based on a semi-structed DSM-5 interview, which was missing for 3 clinical adolescents, estimated IQ scores were missing for 6 clinical adolescents and 15 high-risk adolescents, the Global Assessment of Functioning was missing for 9 clinical adolescents and 2 high-risk adolescents. *Numbers represent % unless otherwise indicated

Supplementary table S2. Differences in severity of the suicidal ideations and number of suicide attempts for the adolescents reporting delusions or hallucinations

	**Suicidal ideation severity**												
	No ideations		*Passive*		*Active, non-specific*		*Active without intent*		*Active, some intent*		*Active, plan and intent*		
	n=137		*n*=16		*n*=19		*n*=13		*n*=25		*n*=35		
	n	%	*n*	%	n	%	*n*	%	*n*	%	*n*	%	
Delusions, non-bizarre ideas													**X^2=52.92, *p*<.001**
absent	129	64.5	11	5.5	16	8.0	11	5.5	18	9.0	15	7.5*	
present	8	17.8***	5	11.1	3	6.7	2	4.4	7	15.6	20	44.4***	
Delusions, unusual thoughts													**X^2=65.36, *p*<.001**
absent	129	70.5	8	4.4	11	6.0	7	3.8	14	7.7	14	7.7*	
present	8	12.9**	8	12.9	8	12.9	6	9.7	11	17.7	21	33.9***	
Hallucinations, perceptual abnormalities													**X^2=31.67, *p*<.001**
absent	119	65.7	11	6.1	12	6.6	7	3.9	16	8.8	16	8.8	
present	18	28.1**	5	7.8	7	10.9	6	9.4	9	14.1	19	29.7***	

	**Number of suicide attempts**						
	None		*One*		*Multiple*		
	n=193		*n*=23		*n*=35		
	n	%	*n*	%	*n*	%	
Delusions, non-bizarre ideas							**X^2=29.27, *p*<.001**
absent	172	83.5%	12	5.8%	22	10.7%	
present	21	46.7%*	11	24.4%***	13	28.9%**	
Delusions, unusual thoughts							**X^2=36.32, *p*<.001**
absent	163	86.2%	9	4.8%	17	9.0%	
present	30	48.4%**	14	22.6%***	18	29%**	
Hallucinations, perceptual abnormalities							**X^2=28.99, *p*<.001**
absent	159	85.0%	9	4.8%	19	10.2%	
present	34	53.1%*	14	21.9%***	16	25%*	

Note. Based on the standardized residual (z-score) significant at * *p*<.05, ** *p*<.01, or *** *p*<.001.

Table S3. Associations between psychotic symptoms and suicidality, including interaction effects: a sex-interaction effect with psychotic symptoms or an interaction effect between hallucinations and any type of delusions (n=251)

	**OR**	**95% CI**		***p*-value**		**OR**	**95% CI**		***p*-value**
Suicidal ideation vs. no suicidality					Suicidal ideation vs. no suicidality				
Intercept				0.62	Intercept				0.47
Sex (female)	1.22	0.51	2.90	0.66	Sex (female)	1.23	0.57	2.65	0.59
Age	0.86	0.71	1.06	0.15	Age	0.84	0.69	1.03	0.10
Delusions, non-bizarre ideas	1.67	0.51	5.45	0.40	Delusions, non-bizarre ideas	1.20	0.30	4.78	0.80
Delusions, unusual thoughts	**7.95**	**2.36**	**26.76**	**0.00**	Delusions, unusual thoughts	**4.75**	**1.19**	**18.94**	**0.03**
Hallucinations, perceptual abnormalities	0.83	0.26	2.66	0.75	Hallucinations, perceptual abnormalities	0.59	0.16	2.17	0.43
Interaction term sex * psychotic symptoms	1.14	0.26	4.99	0.87	Interaction term hallucinations * delusions	3.36	0.41	27.42	0.26
Suicide attempt vs. no suicidality									
Intercept					Intercept				0.53
Sex (female)	**3.13**	**1.09**	**8.99**	**0.03**	Sex (female)	**3.81**	**1.50**	**9.68**	**0.00**
Age	0.94	0.76	1.16	0.56	Age	0.93	0.75	1.15	0.48
Delusions, non-bizarre ideas	**3.27**	**1.07**	**9.93**	**0.04**	Delusions, non-bizarre ideas	**3.36**	**0.95**	**11.93**	**0.06**
Delusions, unusual thoughts	**5.31**	**1.72**	**16.40**	**0.00**	Delusions, unusual thoughts	**5.46**	**1.42**	**21.00**	**0.01**
Hallucinations, perceptual abnormalities	1.29	0.40	4.14	0.67	Hallucinations, perceptual abnormalities	1.54	0.45	5.19	0.49
Interaction term sex * psychotic symptoms	1.83	0.43	7.76	0.41	Interaction term hallucinations * delusions	1.47	0.20	10.66	0.70

Note. Absence of suicidality was the reference category.

Supplementary table S. Differences in psychotic symptoms and reported suicidality in 251 adolescents, stratified by cohort

	**High-risk cohort (n=156)**							**Clinical cohort (n=95)**						
	Suicidality							Suicidality						
	None		*Ideations*		*Attempts*			*None*		*Ideations*		*Attempts*		
	n=116		*n*=22		*n*=18			*n*=26		*n*=28		*n*=41		
	n	%	*n*	%	*n*	%		*n*	%	*n*	%	*n*	%	
Delusions, non-bizarre ideas							**Fisher=12.66, *p*<.001**							**Fisher=9.39, *p*=.008**
absent	111	77.1	21	14.6	12	8.3		23	37.1	17	27.4	22	35.5	
present	5	41.7	1	8.3	6	50		3	9.1	11	33.3	19	57.6	
Delusions, unusual thought content							**Fisher= 15.00, *p*<.001**							**Fisher=14.55, *p*<.001**
absent	114	78.1	18	12.3	14	14.1		20	46.5	10	23.3	13	30.2	
present	2	20	4	40	4	11.5		6	11.5	18	34.6	28	53.8	
Hallucinations, perceptual abnormalities							**Fisher=9.73, *p*=.004**							Fisher=1.45, *p*=.493
absent	110	75.9	22	15.2	13	9.0		14	33.3	12	28.6	16	38.1	
present	6	54.5	0	0.0	5	45.5		12	22.6	16	30.2	25	47.2	

Supplementary table S5. Coefficients from multinomial regression analyses for the association between psychotic symptoms and suicidality, absence of suicidality is the reference category

	**High-risk cohort (n=156)**				**Clinical cohort (n=95)**			
	OR	95% CI		*p*-value	OR	95% CI		*p*-value
Suicidal ideation vs. no suicidality								
Intercept				.88				.18
Sex (female)	1.20	0.46	3.14	.70	0.77	0.17	3.45	.74
Age	0.95	0.51	1.75	.86	1.28	0.86	1.89	.23
Delusions, non-bizarre ideas	0.49	0.04	5.75	.57	2.76	0.55	13.83	.22
Delusions, unusual thoughts	**13.93**	**2.17**	**89.53**	**.01**	**5.07**	**1.29**	**19.94**	**.02**
Hallucinations, perceptual abnormalities	---	---	---	---[1]	0.83	0.23	3.01	.77
Suicide attempt vs. no suicidality								
Intercept				.47				.02
Sex	2.25	0.73	6.99	.16	8.86	0.81	97.14	.07
Age	1.11	0.56	2.20	.77	1.46	0.97	2.18	.07
Delusions, non-bizarre ideas	**7.09**	**1.64**	**30.68**	**.01**	3.79	0.81	17.79	.09
Delusions, unusual thoughts	**7.06**	**0.99**	**50.14**	**.05**	**5.70**	**1.53**	**21.22**	**.01**
Hallucinations, perceptual abnormalities	---	---	---	---[1]	0.77	0.22	2.70	.68

Note. [1] In the high-risk cohort the multinomial regression did not converge because of separation in the hallucination variable, a simplified model without the hallucination variable is presented.
